# David S. Saunders: man of insects and photoperiodism (1935–2023)

**DOI:** 10.1007/s00359-023-01665-3

**Published:** 2023-08-06

**Authors:** Charlotte Helfrich-Förster

**Affiliations:** Neurobiology and Genetics, Biocentre, University of Würzburg, Am Hubland, 97074 Würzburg, Germany

**Keywords:** Circadian clocks, Photoperiodism, University of Edinburgh, Erasmus Summer School Chronobiology, Passion for nature and science, David S. Saunders

## Abstract

David S. Saunders was an outstanding scientist, who devoted his life to his family and to insects. He has made many fundamental contributions to our understanding of how insects reproduce and adapt their reproduction and development to the seasonal changes on our planet. Most importantly, he was a pioneer in demonstrating the role of the circadian clock in insect photoperiodic time measurement, first in the jewel wasp *Nasonia vitripennis*, and later in varies species of flies. His books on biological rhythms and insect clocks are important undergraduate, graduate and research reference literature. David was also a brilliant teacher and mentor and played a major role in establishing and teaching a series of successful Erasmus-funded Chronobiology Summer Schools in Europe. He leaves behind a legacy, both professionally and personally.

**Introduction**  David Stanley Saunders was a pioneer in insect physiology, chronobiology and photoperiodism, a passionate researcher, beloved colleague, trusted friend, and enthusiastic teacher. He died on April 22, 2023, about one month after his 88th birthday following increasing health problems that come with age. David enjoyed life at the fullest and leaves behind a lasting legacy, both professionally and personally. He was an exceptionally talented chronobiologist, and his scientific work was admired across the world. David continued scientific writing right up to the end of his distinguished life, and just before he passed away, he finished his last review article, which can be found in this special issue of the Journal of Comparative Physiology A (Saunders 2023).

## Personal life

David was born on March 12, 1935, and grew up in Pinner, Middlesex. Already as a boy, he developed a strong interest in entomology, which was promoted by his father John Michael Kingsley Saunders; and together with his father, he became a specialist in butterflies and moths. This early passion for insects laid the ground for his later successful scientific career. David’s second life-time passion was cycling. In 1949, at the age of 14, he joined, together with a close friend, the local cycling club, the Northwood Wheelers. This proved to be character building and allowed him to develop autonomy and freedom in the difficult years after the Second World War. He wrote cycling diaries and monitored the miles he had cycled on his many trips including those to work: 160,000 miles altogether—more than half the distance from the Earth to the Moon. For his undergraduate studies, David went to King’s College in London to read Zoology. After having obtained his first-class honours BSc degree, he started his doctoral studies on tsetse flies and the parasitic wasp *Nasonia vitripennis* at the London School of Hygiene and Tropical Medicine and got his Doctorate in 1958, just 2 years after he gained his BSc.

Already as a PhD student David met his later wife Jean, who worked as a secretary in the Institute. Six weeks after they had met, David and Jean became engaged, and in 1959 they married —a marriage that would last 58 years and be extraordinarily happy until Jean’s sad death in 2017. Jean was incredibly supportive of David’s career and activities, and they were tremendously close to each other. David was very much a family man. He was devoted to Jean and a terrific father to the three sons, Robert, Michael, and Richard. He took them out on camping trips, introduced them to exotic food, occasionally delighted them with local trips to sweet shops and many nature excursions, including bike trips. From time to time, David took them to the Institute. At that time, he worked on diapause in flesh flies and the sons could watch the larvae growing on rotten meat. Although the smell was disgusting, the boys were fascinated by the moving animals and their biology. For half a year, the entire family accompanied David on his first sabbatical at Stanford University, California (1971–1972). David installed in his sons a deep interest in science. All three followed their father’s footsteps and became professional biologists. After they had left the nest, and particularly during his retirement, David and Jean travelled extensively all over the world, often combined with scientific meetings or with visiting friends they had made over the years. They have been at the Iguacu Falls in Brazil, Petra in Jordan, in Morocco, Uganda, Nigeria, and different places in the USA and Europe. They visited their son Richard and his family in Hong Kong six times. In later years, David was very proud of his three grandchildren, Arran, David, and Ines, speaking regularly to his family by Facetime, keeping up with and interested in their lives and their various achievements.

David was quite an adventurer. As a student, he travelled to remote parts of Africa, such as Uganda, Nigeria, Burkina Faso, by train, bus, boat, and plane with a return via Nile valley, Sudan and Egypt. As a young lecturer he participated in a University of Edinburgh student expedition that involved travelling in Land Rovers through Europe and Turkey to Iran. During this expedition he collected ticks for the University. Later, as chairman of the Edinburgh University expedition committee, he became involved in an expedition to newly discovered caves in Ecuador. He went there in a scouting expedition that involved travelling via Newfoundland, Miami, Nassau, Jamaica, Panama and Colombia before finally arriving in Quito, Ecuador. On the return trip, as well as during one of his multiple trips to the US, he bought a hundred-day Greyhound Bus pass and used this extensively for travelling through the US, often sleeping in the bus.

## Scientific career

In 1958, David was appointed assistant lecturer at the Department of Zoology in Edinburgh. He remained at the University of Edinburgh for almost his entire career, except for three visiting professorships, first at Stanford University, California (1971–1972) and then twice at the University of North Carolina at Chapel Hill (1983 and 1987–1988).

In 1990, David was promoted to Professor of Insect Physiology at the Department of Zoology in Edinburgh and 3 years later he became the Head of the Institute of Cell, Animal and Population Biology. He held this position until 1997 and was fair and impartial in all his actions in the administration, although he did not enjoy this activity. In truth, he was first and foremost a researcher and teacher.

David’s excellent contributions to the scientific field have been recognised around the world with prizes and honours, such as the Patrick Buxton Memorial Essay Prize, the Medal of the Polish Physiological Society in 1990 and his election as a Fellow of the Royal Society of Edinburgh in 1995. He continuously held grants from different funding agencies and was regularly invited to international scientific conferences.

David officially retired from the University of Edinburgh in 1999. In honour of his retirement, a farewell party was held in form of the ‘Complex Clock’ Conference, in March 2000. This international meeting attracted many of his friends and colleagues (including myself) to review and discuss recent developments in the field of biological timing. The scientific contributions to this conference were published in two special collections, the ‘Complex Clocks’ special issue in the *Philosophical Transactions: Biological Science* (2001), and the ‘Complex Insect Clocks’ special issue in the *Journal of Insect Physiology* (2001). Both special issues are an appreciation of David’s academic work, with the latter containing a special tribute to David by Vaz Nunes (2001). Despite his formal retirement, David never retired from science. He wrote more than 50 scientific articles after retirement, of which only his last four review articles are listed here (Saunders 2020, 2021a, 2021b, 2022).

## Scientific work

David’s early work focused on tsetse flies and their pupal parasites, for which he did fieldwork in Africa. In 1960, he described for the first time the precise morphology of the ovaries in *Glossina morsitans* in the scientific journal *Nature* (Saunders 1960a). He found that each ovary contains two ovarioles, in which one follicle after the other develops in a defined order. This paper challenged older work that reported only one ovariole in each ovary of the tsetse fly, and, thus, had important consequences for our understanding of the ovulation cycle of tsetse flies. Unlike other flies, viviparous tsetse flies produce very few offspring and need several blood meals to do so. Well-fed flies produce single larva at about ten-day intervals from eggs ovulated alternately from left and right ovaries, and the changing appearance of their ovaries can be used for determining their physiological age (Saunders 1960b, c, 1961a). David extended his investigations to other blood-sucking fly species (Saunders 1962, 1964a), and at the same time delved deeply into their pupal parasites as well as those of other flies (Saunders 1957, 1960d, e, f, 1961b, 1964b). The work on the jewel wasp, *Nasonia vitripennis*, which parasitises the pupae of blow flies, led David finally to the phenomenon of diapause. He found that the *Nasonia* larvae stopped developing under short days and that the timing of this arrest (diapause) was determined by the photoperiodic experience of the mother wasp (Saunders 1965a, b, 1966a, b).

This discovery not only led to his third *Nature* paper but turned his interest to photoperiodic events in the lives of insects—a field to which he has made major contributions. He was one of the first to demonstrate the role of the circadian clock in insect photoperiodic time measurement, first in the jewel wasp *Nasonia vitripennis* and later in the flesh fly *Sarcophaga argyrostoma*, and the blow fly *Calliphora vicina* (Saunders 1967, 1968, 1970, 1971). During his sabbatical with Colin Pittendrigh at Stanford University, California (1971–1972), David became familiar with the internal and external coincidence models of diapause control by the circadian clock, and he demonstrated that the jewel wasp follows internal coincidence while the flies rather follow external coincidence (Saunders 1974, 1978a, 1979a, 1982a, 1984, 1992, see also Saunders, this issue). He also studied the endocrinology of diapause using *Calliphora vicina*, *Sarcophaga argyrostoma* and *Drosophila melanogaster* as examples of larval, pupal and adult reproductive diapause, respectively (Saunders 1980, 1981a, b; Giebultowicz and Saunders 1983). Furthermore, he achieved the first in vitro reprogramming of the photoperiodic clock in an insect brain-retrocerebral complex in the tobacco hornworm *Manduca sexta* (Bowen et al. 1984).

At the time, however, there was a lively debate about whether the circadian clock is involved in photoperiodism at all, since aphids and spider mites do not seem to use the circadian clock but an hourglass as an internal reference timer for determining daylength. David elegantly solved this discrepancy by playing around with mathematical models and by showing that an hourglass can be regarded as highly dampened circadian oscillator (Lewis and Saunders 1987; Saunders and Lewis 1987a, b, 1988). Different insects, therefore, appear to possess circadian clocks with different degrees of dampening. While the circadian clock of blow flies is rather strong, showing merely no dampening, the aphid and spider mite clocks dampen within one day and look like an hourglass. Most importantly, however, the damping does not interfere with the measurement of daylength because during the daily light-dark cycles a damped clock is just as good a reference timer as a strong clock.

Still, the fruit fly *Drosophila melanogaster* puzzled David’s theory. With the aim of testing the genetic basis of the photoperiodic response, David investigated the photoperiodic response of *period* mutants with fast (*per*^*s*^), slow (*per*^*l*^) and no (*per*^*0*^) clock. He expected that the short- and long-period mutants would show a shift in the critical daylength at which they would go into diapause, while the arrhythmic mutants should no longer be able to measure daylength. However, this was not the case. He observed only slight differences in the critical daylength in the short and long *period* mutants, and the mutants without functional circadian clocks were still able to measure daylength almost normally (Saunders et al. 1989; Saunders 1990). This discrepancy is still not completely solved, although David explained it by a coupled pacemaker-slave system (Gillanders and Saunders 1992). Coupled oscillators might represent a general model for the photoperiodic clock in insects (Vaz Nunes et al. 1991a, b; Nunes and Saunders 1999).

In the last decade of the twentieth century, David’s research interests focused on determining the anatomical location of circadian and photoperiodic photoreceptors and circadian clocks, which led to a series of experiments with *Calliphora vicina*, involving a collaboration with colleagues from Warsaw, North Carolina, Auckland and Taiwan (Cymborowski et al. 1994, 1996; Saunders and Cymborowski 1996, 2003; Hong and Saunders 1998).

Research was David’s greatest passion, to which his bibliography bears witness. He has been one of the pioneers of chronobiology worldwide: his work has resulted in over 190 publications including three books and 41 book chapters. His first book “An Introduction to Biological Rhythms” for university students appeared in 1977 (Saunders 1977), was translated into Japanese (Saunders 1978b) and revised in 1979 (Saunders 1979b). His classic book “Insect Clocks” appeared in four editions (Saunders 1976, 1978a, b, Saunders 1982b, 2002). Both books are still important reference literature for teaching and research. In 2001, David published, together with David Denlinger and Jadwiga Giebultowicz, a third book about “Insect Timing: Circadian Rhythmicity to Seasonality” (Denlinger et al. 2001). In December 2022, he wrote the Preface for the book “Insect Chronobiology” edited by Numata and Tomioka (2023).

### Teaching and mentoring

Throughout the years David had been an excellent and committed teacher. Many students have graduated with doctorates under his enthusiastic guidance and supervision. He also played an important role in setting up and running several successful Erasmus-funded Chronobiology Summer Schools in Edinburgh and other universities in Europe. As a scientific colleague, David was always generous and helpful, as anyone who has worked with or met him can testify.

**Fig. 1:**
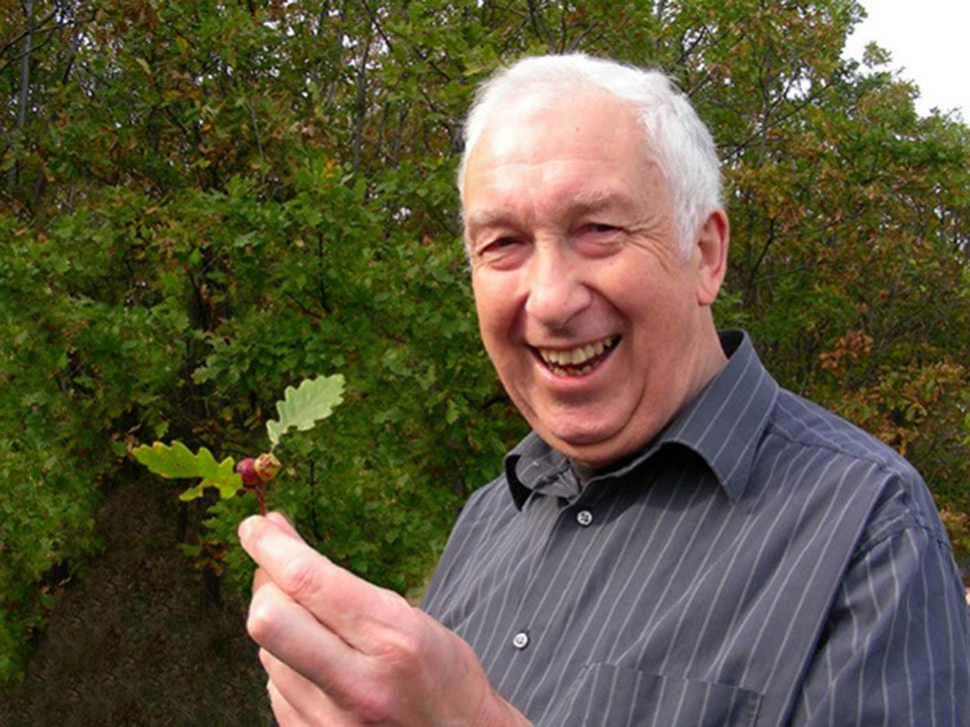
David Saunders at the Erasmus Summer School on Chronobiology in Matrahaza, Hungary, in 2007. (Photograph: Charlotte Helfrich-Förster.)

I met David for the first time at an Erasmus Summer School in Groningen in 1991. At that time, I was a postdoc in Wolfgang Engelmann’s lab (Tübingen) and very much interested in photoperiodism. David’s article about photoperiodic responses in the arrhythmic *per*^*0*^ mutants of *D. melanogaster* had just been published, and this paper questioned the view that circadian clocks are needed for photoperiodic responses (Saunders et al. 1989; Saunders 1990). At the same time, I had gained some evidence that *per*^*0*^ mutants still have a residual circadian clock, and I wanted to test whether this residual clock is responsible for their photoperiodic responses. I approached David and told him about my ideas of how to solve this question. He listened with great patience, and then he advised me not to carry out my plans. I still remember his words: “Don’t do it! Fruit flies have such a shallow diapause that you will not get clear results.” Although disappointed to some degree, I followed his advice and decided to first resolve the anatomy of *D. melanogaster*’s circadian clock, its photoreceptor input, and its connection to the neurohormonal system before approaching again the question about the role of the clock in photoperiodic responses. Now, after more than 30 years, I am at the point to delve again in photoperiodism, and in retrospect I am very grateful for David’s advice. David and I met at several further occasions including the above mentioned ‘Complex Clocks’ Conference in honour of David’s formal retirement. David was always very interested in my scientific progress, while I continued to esteem his scientific guidance. The last time we met personally was again at an Erasmus Summer School, which took place in Matrahaza, Hungary in 2007. David very much enjoyed an excursion to the local woods and immediately found several biological objects, which he enthusiastically demonstrated to the students. Figure 1 shows him with oak leaves carrying nutgalls that were produced by a gall wasp.

**Fig. 2:**
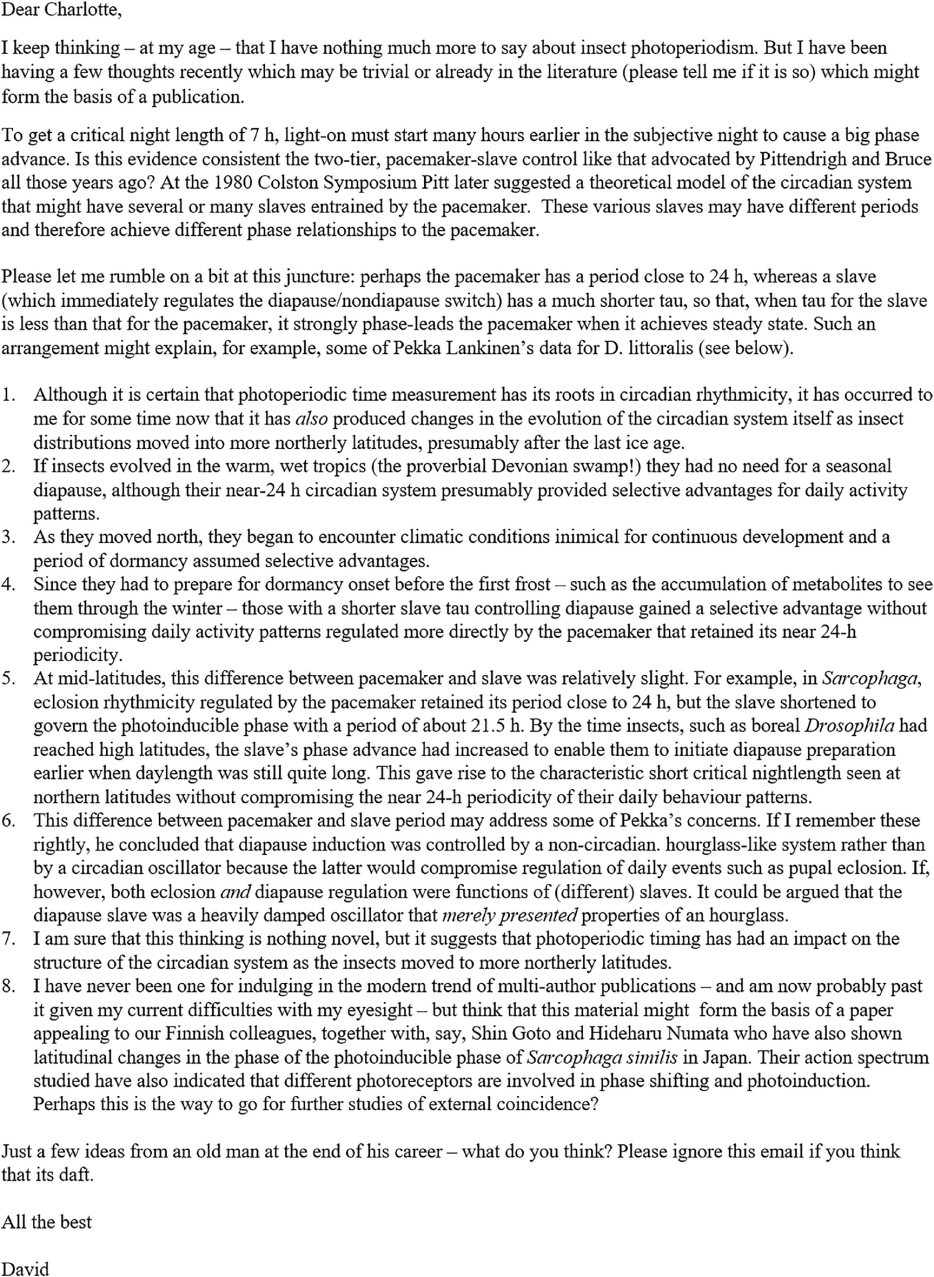
Email from David Saunders written on March 20, 2023, about his thoughts on the involvement of the circadian clock in photoperiodic responses, which as he suggested might be the basis of a future multi-author publication of scientists from Finland and Japan

Although we did not meet again, David and I stayed in loose contact, and I followed all his review articles with great interest. In autumn 2022, I asked him whether he would be interested in contributing to a planned special issue “A clock for all seasons”, and I was delighted that he agreed. From that point on we had rather regular scientific exchanges via e-mail, and in March 2023, we started a vivid discussion about circadian pacemakers and slaves and about how insects changed their circadian system while their distributions moved into more northerly latitudes. He tried to find a scientific solution to the phenomenon that the critical nightlength for entering overwintering diapause becomes shorter with increasing latitude, but the circadian pacemaker underlying timekeeping changes much less. I would like to share one of his last emails with the scientific community (Fig. 2). This email is a testament to David’s passion for insect biology and in particular insect photoperiodism.

Short reminiscences from a few of David’s colleagues may add to the depth of his natural curiosity in all things concerning biology and his passion for science.

Christina Holzapfel and William Bradshaw (University of Oregon, US). *“We smiled as we recalled David’s visit to Oregon some 15 years ago, when we took him to the coastal high dunes, white sand beaches, and intertidal rockpools. He was an enthusiastic hiker in the dunes, and enchanted with the diversity of intertidal life. He was a joy to behold: pant legs rolled up, scampering into the intertidal boulder field like a child in a toy store, turning over rocks, combing through beds of seaweed, chasing after retreating crabs, and generally splashing about. We finally had to threaten him with abandonment on the shore if he would not return to the car as dusk settled upon us. Such was David Saunders, the consummate naturalist!”*

David Dolezel (BCAS, Ceske Budejovice, Czech Republic). *“I met David Saunders in 2011, and it was one of the most inspiring meetings of my life. At the time, David was 76 years old and had been retired for more than a decade. Yet, David was an amazingly vital person who was passionate about wildlife and science. Not only was he brightly discussing anything, but he apparently knew all the current literature, including papers that had been published only a week ago. This man embodied pure passion and curiosity.“*.

Charalambos Kyriacou (University of Leicester, UK). „*David’s irreverent humour is very much to the fore in the last email he wrote to me: Dear Bambos, just to let you know that this old f.t is still alive - although his 85th birthday looms. I still like to keep abreast of things - my recent review is attached - mainly to keep the brain active. It is now two years since Jean died, and writing that review kept me sane. My three sons are scattered: Robert comes up to see me and drink my beer very frequently; otherwise Michael is in Brussels and Richard in Hong Kong, and I see less of them. I try to keep up with the Drosophila diapause story. I see from your 2018 paper (Anduaga et al. JIP 105:46–53) you mention work by Nagy (in press) on flies in natural light. Has that appeared? Have I just missed it? I would much appreciate it if you could keep me in the loop. If you are ever back in Auld Reekie, you know where I live. David“*.

The world has lost a great scientist, and many of us have lost a dear colleague, mentor, and friend. It is a comfort that David’s influence in the world remains, and it will continue to flow as a consequence of his character and his actions.

## References

[CR1] Bowen MF, Saunders DS, Bollenbacher WE, Gilbert LI (1984). In vitro reprogramming of the photoperiodic clock in an insect brain-retrocerebral complex. Proc Natl Acad Sci U S A.

[CR2] Cymborowski B, Lewis RD, Hong S-F, Saunders DS (1994). Circadian locomotor activity rhythms and their entrainment to light-dark cycles continue in flies (*Calliphora vicina*) surgically deprived of their optic lobes. J Insect Physiol.

[CR3] Cymborowski B, Hong SF, McWatters HG, Saunders DS (1996). S-antigen antibody partially blocks entrainment and the effects of constant light on the circadian rhythm of locomotor activity in the adult blow fly, *Calliphora vicina*. J Biol Rhythms.

[CR4] Denlinger DL, Giebultowicz JM, Saunders DS (2001). Insect timing: circadian rhythmicity to seasonality.

[CR5] Giebultowicz JM, Saunders DS (1983). Evidence for the neurohormonal basis of commitment to pupal diapause in larvae of *Sarcophaga argyrostoma*. Experientia.

[CR6] Gillanders SW, Saunders DS (1992). A coupled pacemaker-slave model for the insect photoperiodic clock: interpretation of ovarian diapause data in *Drosophila melanogaster*. Biol Cybern.

[CR7] Hong S-F, Saunders DS (1998). Internal desynchronisation in blow fly (*Calliphora vicina*) locomotor activity rhythms: evidence for a complex circadian pacemaker. Biol Rhythm Res.

[CR8] Lewis RD, Saunders DS (1987). A damped circadian oscillator model of an insect photoperiodic clock. I. description of the model based on a feedback control system. J Theor Biol.

[CR9] Numata H, Tomioka K (2023). Insect chronobiology.

[CR10] Nunes MV, Saunders DS (1999). Photoperiodic time measurement in insects: a review of clock models. J Biol Rhythms.

[CR11] Saunders DS (1957). Material from a laboratory culture of *Syntomosphyrum glossinae* Waterston, a chalcid parasite of tsetse fly puparia. Trans R Soc Trop Med Hyg.

[CR12] Saunders DS (1960). Ovaries of *Glossina morsitans*. Nature.

[CR13] Saunders DS (1960). The ovulation cycle in *Glossina morsitans* Westwood (Diptera: Muscidae) and a possible method of age determination for female tsetse flies by the examination of their ovaries. Trans R Entomol Soc Lond.

[CR14] Saunders DS (1960). Determination of physiological age for female *Glossina morsitans*. Nature.

[CR15] Saunders DS (1960). Some records of Dipterous and Hymenopterous pariasites of tsetse fly pupae. Proc R Entomol Soc Lond Ser Gen Entomol.

[CR16] Saunders DS (1960). On the stages in the development of *Syntomosphyrum albiclavus* Kerrich (Hym., Eulophidae), a parasite of tsetse flies. Bull Entomol Res.

[CR17] Saunders DS (1960). The “white-clubbed” form of *Syntomosphyrum* (hym., eulophidae) parasitic on tsetse flies. Bull Entomol Res.

[CR18] Saunders DS (1961). Studies on ovarian development in tsetse flies (Glossina, Diptera). Parasitology.

[CR19] Saunders DS (1961). Laboratory studies on the biology of *Syntomosphyrum albiclavus*.Kerrich (Hym. Eulophidae), a parasite of tsetse flies. Bull Entomol Res.

[CR20] Saunders DS (1962). Age determination for female tsetse flies and the age compositions of samples of *Glossina pallidipes* Aust., *G. palpalis fuscipes* Newst. and *G. brevipalpis* Newst. Bull Entomol Res.

[CR21] Saunders DS (1964). Age-changes in the ovaries of the sheep ked, *Melophagus ovinus* (L.) (Diptera: Hippoboscidae). Proc R Entomol Soc Lond Ser Gen Entomol.

[CR22] Saunders DS (1964). Rearing tsetse fly parasites in blowfly puparia. Bull World Health Organ.

[CR23] Saunders DS (1965). Larval diapause induced by a maternally-operating photoperiod. Nature.

[CR24] Saunders DS (1965). Larval diapause of maternal origin: induction of diapause in *Nasonia vitripennis* (Walk.) (Hymenoptera: Pteromalidae). J Exp Biol.

[CR25] Saunders DS (1966). Larval diapause of maternal origin—II. The effect of photoperiod and temperature on *Nasonia vitripennis*. J Insect Physiol.

[CR26] Saunders DS (1966). Larval diapause of maternal origin—III. The effect of host shortage on *Nasonia vitripennis*. J Insect Physiol.

[CR27] Saunders DS (1967). Time measurement in insect photoperiodism: reversal of a photoperiod effect by chilling. Science.

[CR28] Saunders DS (1968). Photoperiodism and time measurement in the parasitic wasp, *Nasonia vitripennis*. J Insect Physiol.

[CR29] Saunders DS (1970). Circadian clock in insect photoperiodism. Science.

[CR30] Saunders DS (1971). The temperature-compensated photoperiodic clock ‘programming’ development and pupal diapause in the flesh-fly, *Sarcophaga argyrostoma*. J Insect Physiol.

[CR31] Saunders DS (1974). Evidence for ‘dawn’ and ‘dusk’ oscillators in the *Nasonia* photoperiodic clock. J Insect Physiol.

[CR32] Saunders DS (1976). Insect clocks.

[CR33] Saunders DS (1977). An introduction to biological rhythms. Tertiary level biology.

[CR34] Saunders DS (1978). Internal and external coincidence and the apparent diversity of photoperiodic clocks in the insects. J Comp Physiol.

[CR35] Saunders DS (1978b) An introduction to biological rhythms, Japanese edition. Rikogakusya and Co, Ltd

[CR36] Saunders DS (1979). External coincidence and the photoinducible phase in the *Sarcophaga* photoperiodic clock. J Comp Physiol.

[CR37] Saunders DS (1979). Insect clocks. Revised students edition.

[CR38] Saunders DS (1980). Some effects of constant temperature and photoperiod on the diapause response of the flesh fly, *Sarcophaga argyrostoma*. Physiol Entomol.

[CR39] Saunders DS, Aschoff J (1981). Insect photoperiodism. Biological rhythms.

[CR40] Saunders DS (1981). Insect photoperiodism — the clock and the counter: a review. Physiol Entomol.

[CR41] Saunders DS (1982). The effects of ultra-short photoperiods on the seasonal clock in *Sarcophaga argyrostoma*. J Comp Physiol.

[CR42] Saunders DS (1982b) Insect clocks, 2nd edn. Pergamon Press, Oxford

[CR43] Saunders DS (1984). Photoperiodic time measurement in *Sarcophaga argyrostoma*: an attempt to use daily temperature cycles to distinguish external from internal coincidence. J Comp Physiol A.

[CR44] Saunders DS (1990). The circadian basis of ovarian diapause regulation in *Drosophila melanogaster*: is the period gene causally involved in photoperiodic time measurement?. J Biol Rhythms.

[CR45] Saunders DS (1992). The photoperiodic clock and “counter” in *Sarcophaga argyrostoma*: experimental evidence consistent with “external coincidence” in insect photoperiodism. J Comp Physiol A.

[CR46] Saunders DS (2002) Insect clocks. Elsevier, Amsterdam

[CR47] Saunders DS (2020). Dormancy, diapause, and the role of the circadian system in insect photoperiodism. Annu Rev Entomol.

[CR48] Saunders DS (2021). Insect photoperiodism: Bünning’s hypothesis, the history and development of an idea. Eur J Neurosci.

[CR49] Saunders DS (2021). A comparative study of circadian rhythmicity and photoperiodism in closely related species of blow flies: external coincidence, maternal induction, and diapause at northern latitudes. J Biol Rhythms.

[CR50] Saunders DS (2022). Time measurement in insect photoperiodism: the role of photophase duration and light intensity. Eur J Entomol.

[CR51] Saunders DS, Cymborowski B (1996). Removal of optic lobes of adult blow flies (*Calliphora vicina*) leaves photoperiodic induction of larval diapause intact. J Insect Physiol.

[CR52] Saunders DS, Cymborowski B (2003). Selection for high diapause incidence in blow flies (*Calliphora vicina*) maintained under long days increases the maternal critical daylength: some consequences for the photoperiodic clock. J Insect Physiol.

[CR53] Saunders DS, Lewis RD (1987). A damped circadian oscillator model of an insect photoperiodic clock: III. Circadian and “hourglass” responses. J Theor Biol.

[CR54] Saunders DS, Lewis RD (1987). A damped circadian oscillator model of an insect photoperiodic clock: II. Simulations of the shapes of the photoperiodic response curves. J Theor Biol.

[CR55] Saunders DS, Lewis RD (1988). The photoperiodic clock and counter mechanism in two species of flies: evidence for damped circadian oscillators in time measurement. J Comp Physiol A.

[CR56] Saunders DS, Henrich VC, Gilbert LI (1989). Induction of diapause in *Drosophila melanogaster*: photoperiodic regulation and the impact of arrhythmic clock mutations on time measurement. Proc Natl Acad Sci U S A.

[CR57] Vaz Nunes M (2001). Professor David S. Saunders: a tribute. J Insect Physiol - J Insect Physiol.

[CR58] Vaz Nunes M, Lewis RD, Saunders DS (1991). A coupled oscillator feedback system as a model for the photoperiodic clock in insects and mites. I. The basic control system as a model for circadian rhythms. J Theor Biol.

[CR59] Vaz Nunes M, Saunders DS, Lewis RD (1991). A coupled oscillator feedback system as a model for the photoperiodic clock in insects and mites. II. Simulations of photoperiodic responses. J Theor Biol.

